# The Current View of Retroviruses as Seen from the Shoulders of a Giant

**DOI:** 10.3390/v11090828

**Published:** 2019-09-05

**Authors:** Jiří Hejnar, Tomáš Ruml

**Affiliations:** 1 Department of Viral and Cellular Genetics, Institute of Molecular Genetics of the Czech Academy of Sciences, Videnska 1083, CZ-14220 Prague, Czech Republic; 2 Department of Biochemistry and Microbiology, University of Chemistry and Technology, CZ-166 28 Prague, Czech Republic

It has now been more than two years since we said our last goodbye to Jan Svoboda (14.8. 1934 –13.3. 2017), a pioneer of retrovirology, brilliant scientist, and for many years a living legend in the broader field of retroviruses, tumor viruses, and oncogenes. Jan’s life and scientific merits have recently been described in two commemorative papers [1,2]. Furthermore, his principal contributions to understanding the genetics of retroviruses and oncogene transduction were put into the context of retrovirus defectiveness research conducted in the 1960s and 1970s in an excellent mini-review [3]. This Special Issue was intended not only to remember Jan’s seminal work, but also to point out the recent progress in topics he pursued during his 60-year long career in virology, and particularly the work he crafted in the last years with his latest students and peers. We believed that two and a half years is a reasonable time to look back and gauge what of these sprouts appears promising and inspirational. In addition, we hoped to assemble contributions from people who were influenced by Jan, either as his students or collaborators, and who now work on different topics of virology all around the world. 

From the very beginning, Jan was fascinated by the problem of cell permissiveness to virus infection and productive replication. His favorite model, Rous sarcoma virus (RSV) and avian sarcoma/leukosis viruses (ASLV) in general, predestined his interest, because it is strictly blocked at several levels in the cells of mammals. The explanation and overcoming of these blocks became a life-long endeavour for Jan, as summarized in his review [4] and one of his last papers [5]. 

Virus entry via specific receptor molecules is an important part of the cell permissiveness, and Jan ignited constant interest in ASLV receptors in his colleagues through the discovery of the receptor for subgroup C RSV [6,7]. This story continued in Prague with the defining of new virus-resistant receptor alleles in chicken lines [8,9,10], discovery of the receptor for the new ASLV subgroup K [11], and formulation of the concept of biotechnological construction of ALV-J-resistant chicken [12,13]. As part of this Special Issue, Mark J. Federspiel [14] published a comprehensive up-dated review on ASLV receptors and introduced the reverse engineering strategy to select mutations in the retroviral envelope glycoprotein that escape the dependence on specific receptors. Examples of such envelope variants that broadened their receptor usage or escaped the antiviral effect of soluble receptor-based immunoadhesines are shown in two other papers of this Special Issue [15,16]. It seems that such escape mutants are less efficient in virus replication and form particles less stable in the extracellular environment [17], features that can be attributed to structural destabilization of the surface envelope subunit and partial priming of the fusogenic moieties localized in the transmembrane subunit. It will be interesting to follow this direction and see whether this is a natural mechanism of ASLV-host coevolution or even a general principle valid for other enveloped viruses. 

The next step of the retrovirus replication cycle is integration of proviral DNA into the host cell genome. Although the target site preference of ASLV is relatively weak, the final provirus localization predicts its transcriptional activity and epigenetic marks deposited at the long terminal repeats. In heterologous mammalian host cells, the suppressive epigenetic effects have been analyzed in Jan’s laboratory [18,19,20,21] as an integral part of the cell permissiveness phenomenon. The description of epigenetic interplay at the site of provirus integration also appeared in this Special Issue: Lam and Beemon [22] described erased DNA methylation at the common integration site of an avian retrovirus and Miklík et al. [23] compared various retroviral vectors for their epigenomic features predicting the proviral transcriptional activity. 

Epigenetic proviral silencing gathered substantial attention in the last decade after Kauder et al. [24] and Blazkova et al. [25] discovered the role of DNA methylation in HIV-1 transcriptional inactivation and latency. Since then, there has been much debate about how to revert HIV-1 latency and diminish the reservoir of latent retroviruses. The concept of “shock and kill’’ strategies in an attempts to cure HIV infection is summarized in a review article by Darcis et al. [26]. In 2002, the data mining paper from Jan’s group [27] predated the genome-wide view on HIV integration [28,29]. Today, the integration site analysis is an indispensable aspect in the research of HIV latency and rebound of infection from the reservoir, as shown in this Special Issue – in contrast to the “shock and kill’’ strategy, integration site selection inspires the “block and lock’’ approach to the functional cure of HIV infection [30]. 

In solving the problem of cell permissiveness, Jan Svoboda stuck to simple retroviruses such as ASLV, but it is much more complex in HIV and other retroviruses harboring the auxiliary genes. These genes were characterized as viral agents counteracting the cellular restriction factors. The cell permissiveness to a given retrovirus thus requires a balance of cellular factors that support virus replication and viral factors interfering with the cellular blocks of virus replication. Trafficking and assembly of retroviruses have not been traditionally viewed from the permissiveness perspective, but cellular factors play important roles in these steps of virus replication. BCA3, which is incorporated into HIV-1 particles by its ability to interact with PKAc may be one of such factors, as shown in Rumlová et al. [31]. The paper by Grznárová-Prokšová et al. [32] contributes to our understanding of targeting the preassembled D-type immature particles to the plasma membrane and acquisition of the Env glycoproteins during this vesicle-mediated process. The restriction factors are represented by SAMHD1 and its role in the down-regulation of HIV dynamics in macrophages [33]. The specific mechanisms governing the HIV latency in plasmacytoid dendritic cells are presented by Font-Haro et al. [34]. 

Endogenous retroviruses are also involved in the cell permissiveness; on the one hand, some remnants of proviral sequences interfere with de novo infections, on the other hand, endogenous retroviruses can complement the defective variants of their exogenous counterparts. Jan’s Prague school pursues the epigenetic regulation of syncytins, human endogenous retroviruses that mediate cell-to-cell fusion in placental trophoblast [35,36,37]. Genome-wide studies of human endogenous retroviruses started in our lab immediately after the first releases of the human genome project [38], and the current progress in bioinformatic identification of fossil proviral copies in the vertebrate genomes [39,40,41] is also exemplified in this Special Issue [42] with the evolutionary analysis of endogenous deltaretroviruses. 

Another constant throughout the scientific career of Jan Svoboda was the use of the chicken as a model for *in vivo* experiments with ASLVs. Several oncogenes such as *src*, *myc*, *myb*, *rel*, *jun*, and *erb* were discovered as tumor-inducing genes transduced by retroviruses. The mechanisms of cell transformation, recombination with retroviruses, oncogene activation/up-regulation, and chronic oncogenesis by retrovirus insertion are topics, which continue to be of interest to virologist [43]. The Prague inbred lines of chickens, originally bred for studies of immune tolerance and immunogentics [44], are still a fruitful tool that is available to an increasing community of poultry researchers. Jan would be glad to see the current progress in chicken genomics and gene manipulation made by his former collaborators and disciples. The up-dated chicken genome assembly and RNAseq databases have enabled us to identify genes in GC-rich regions, which used to be declared as missing in the chicken genome [45]. Some of these genes are important factors of the immune system, such as tumor necrosis factor α [46]. The new facets of the innate immune system in the chicken help to specify the host response to viral infection, as shown in this Special Issue as well [47].

The chicken as a model organism has long suffered from the absence of reverse genetics, whose tools are quite common in the mouse and other laboratory animals. The unique reproduction strategy of birds claimed the use of primordial germ cells for introducing gene knock-out in the chicken genome [48]. Because this technique was enormously laborious, it did not produce further knock-outs until recently, when the primordial germ cells were applied by orthotopic transplantation into adult animals [49], which made the procedure much easier and more efficient. From the point of view of retrovirology, the suitable targets to be knock-outed are receptors for ASLVs, depletion of which can lead to chicken resistance to avian leukosis. *In vitro* results suggest that this goal is achievable [13].

Jan Svoboda is also scientifically survived by his son, Jan Svoboda Jr., who focuses on the 20th century philosophy, particularly on Whitehead’s philosophy and what is called the Czech positivism. This was a lucky opportunity for Jan to have a guide and assistance in formulating his own philosophical approach to the methodology of science and critical thinking in biology. Reading the brief application of the Platonian receptacle on genome evolution [50] opens a new view of Jan, who was not only a gifted laboratory scientist, but also a broad-minded thinker, impassionedly precipitating knowledge from the unknown.

We thank the authors and reviewers for their efforts when contributing to this collection of articles. We believe that the Special Issue has provided a fresh insight into the many facets of recent retrovirus research and will inspire future questions, ideas, and experiments.
